# Numerical Study of Concrete Mesostructure Effect on Lamb Wave Propagation

**DOI:** 10.3390/ma13112570

**Published:** 2020-06-04

**Authors:** Beata Zima, Rafał Kędra

**Affiliations:** Department of Mechanics of Materials and Structures, Faculty of Civil and Environmental Engineering, Gdańsk University of Technology, Narutowicza 11/12, 80-233 Gdansk, Poland; rafal.kedra@pg.edu.pl

**Keywords:** concrete, mesostructure, Lamb wave, heterogeneity, Monte Carlo method, SHM

## Abstract

The article presents the results of the numerical investigation of Lamb wave propagation in concrete plates while taking into account the complex concrete mesostructure. Several concrete models with randomly distributed aggregates were generated with the use of the Monte Carlo method. The influence of aggregate ratio and particle size on dispersion curves representing Lamb wave modes was analyzed. The results obtained for heterogeneous concrete models were compared with theoretical results for homogeneous concrete characterized by the averaged macroscopic material parameters. The analysis indicated that not only do the averaged material parameters influence the dispersion solution, but also the amount and size of aggregate particles. The study shows that Lamb waves propagate with different velocities in homogeneous and heterogeneous models and the difference increases with aggregate ratio and particle size, which is a particularly important observation for wave-based diagnostic methods devoted to concrete structures.

## 1. Introduction

Ultrasonic waves have been widely used in diagnostics of engineering structures. Significant scientists’ interest has been focused on the nondestructive evaluation of concrete, which is one of the most popular construction materials used in the world. Its widespread use entails the need for the development of effective methods of damage detection in the early stage to prevent the development of significant defects, which would jeopardize the integrity and safety of the entire structure.

So far, a number of various approaches devoted to solving different problems have been proposed. Ultrasonic waves are most commonly used for crack detection [1,2,3,4,5,6,7], monitoring the hardening process [8,9,10,11], as well as for the quality assessment of the adhesive connection between concrete cover and reinforcement [12,13,14,15,16]. The method of scanning the surface opening cracks in reinforced concrete structures using transient elastic waves was developed by Liu et al. [1]. The effect of the depth of surface-breaking cracks in concrete plates on Lamb wave propagation was described by Yang et al. [2]. The influence of the width of partially closed surface-breaking cracks in concrete structures, the incident angle of waves with cracks, and the distance from the cracks on travel time and wave amplitude have been investigated by Pahlavan et al. [3]. Ultrasonic shear-wave tomography was used by Choi et al. [4] to identify horizontal cracks or delamination in concrete pavements, columns, and bridges. Surface wave propagation was used by Ham et al. [5] to assess the volume content of relatively small distributed defects and to characterize the microcrack networks in concrete. A combination of Rayleigh and longitudinal waves was employed by Aggelis and Shiotani [6] to evaluate the parameters of surface opening cracks in concrete before impregnation by the epoxy material, as well as to determine the final repair effectiveness. Quiviger et al. [7] conducted a simulation study of the influence of the depth and morphology of cracks in concrete on ultrasound diffusion.

Wave propagation was also effectively employed to monitor the concrete hardening process. The speed of propagation of ultrasound in various concrete samples varying in time of aging was investigated by del Rio et al. [8]. Piezoelectric transducers embedded in concrete structures were used by Dumoulin et al. [9] to monitor the setting and hardening phases of the early-age concrete. The hardening process of ultra-high-performance concrete was monitored by Lee et al. [10] using the characteristics of individual Lamb wave modes. The correlation between the shear wave velocity and penetration resistance for mortar mixtures was presented by Liu et al. [11].

Several scientific papers were devoted to guided waves for the detection of debonding occurring between concrete and internal or external reinforcement [12,13,14,15]. Monitoring of the interfacial debonding of a concrete-filled pultrusion-GFRP tubular column based on stress wave propagation was conducted by Yang et al. [12]. The wavelet packet-based energy index was proposed by Jiang et al. [13] to detect debonding between a steel beam and a carbon fiber-reinforced polymer plate. The time-reversal method was applied by Zhao et al. [14] to detect and localize the debonding along the steel–concrete interface. Giri et al. [15] employed the partial least-squares regression technique for detecting gaps in the steel–concrete composite specimens.

The above brief review of the literature indicates the multiplicity of investigation strands concerning wave-based concrete diagnostics carried out in the last decades. However, in the majority of reported cases, regardless of the considered problem, the diagnostic process requires some reference data collected for an intact structure, which would be compared to the data obtained for the damaged specimen. Due to obvious reasons, it is not always possible to make the baseline measurement for a pristine structure, and thus, the development of reference-free diagnostic procedures is of particular importance. The main idea of the recently developed reference-free methods is based on the comparison of the experimental measurement with the theoretical predictions [16]. The greater the difference between experimental and theoretical results, the greater the state of deterioration is expected. However, the discrepancies between experimental measurements and theoretical predictions were caused not only by the damage occurrence but also the simplification of the theoretical model of wave propagation in the concrete structure. The theoretical models were usually developed for homogeneous, isotropic materials characterized by the averaged macroscopic parameters, while the concrete is a multi-phase, strongly heterogeneous material. The complex mesostructure of the concrete led to inaccuracies in the wave propagation model. The theoretical predictions differed slightly from the actual results registered for experimental specimens, which, in consequence, led to inaccuracies in damage size assessment.

Although the papers published by Xu et al. [17], Abo-Quadis [18], and Ramaniraka et al. [19] deal with the impact of the concrete mesostructure on waves characteristics, the problem of wave propagation in heterogeneous materials is still not considered in detail, yet. The existence of aggregates varying in size, shape, and material parameters, and pores and cracks may lead to additional disturbing phenomena like scattering, diffractions, and wave attenuation affecting wave amplitude or propagation velocity, which were commonly used as indicative parameters in the above-mentioned studies. The wave propagation phenomenon may significantly differ for two different concrete specimens despite their identical macroscopic material parameters. Thus, the comprehensive analysis of wave propagation in concrete specimens taking into consideration their complex mesostructure is crucial for the further development of wave-based concrete diagnostic methods.

The main contribution of the paper is the analysis of the aggregate size and content on Lamb wave propagation. The algorithm of the generation of heterogeneous concrete models was developed and described step by step. Several numerical plate models differing in parameters of the internal mesostructure were developed using the Monte Carlo method. The averaged macroscopic parameters determined theoretically were compared to material parameters determined based on the shape of reconstructed dispersion curves for antisymmetric Lamb modes. The results obtained show that the concrete mesostructure has a significant influence on the wave propagation phenomenon. The wave velocity determined for the homogeneous concrete model differed from the velocity in the heterogeneous concrete model, even if the averaged material parameters were the same, and according to the theoretical wave propagation model, differences in velocities should not be observed. The difficulties in exact wave velocity determination must be taken into account, especially if the developed diagnostic procedure involves monitoring of wave propagation velocity.

## 2. Theoretical Background

### 2.1. Theory of Lamb Waves

**Figure 1:**
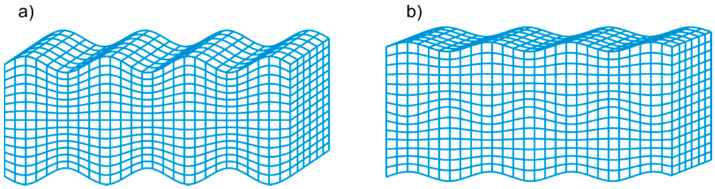
Lamb wave modes: (**a**) Symmetric mode; (**b**) antisymmetric mode.

The existence of Lamb waves was described by Horace Lamb in 1917 [20]. Lamb waves are guided between two parallel free surfaces and can thus be sustained in thin plates. Due to the dispersive nature of Lamb waves, their velocity and number of wave modes depend on excitation frequency. In general, two different mode families can be distinguished: Symmetric and antisymmetric wave modes. When the wave motion is associated with symmetrical displacement and stresses with respect to the middle plane, the symmetric wave mode propagates (Figure 1a). Antisymmetric displacements and stresses are associated with antisymmetric modes propagation (Figure 1b). The relationship between wavenumber and frequency for symmetric modes and antisymmetric modes can be obtained by solving the following dispersion equations, respectively:

(1) $$\frac{\tan\left(qd\right)}{\tan\left(pd\right)}=-\frac{4k^{2}pq}{{\left(k^{2}-q^{2}\right)}^{2}}$$

(2) $$\frac{\tan\left(qd\right)}{\tan\left(pd\right)}=-\frac{{\left(k^{2}-q^{2}\right)}^{2}}{4k^{2}pq}$$

where *k* is the wavenumber, *d* is specimen thickness, and *p* and *q* depend on angular frequency *ω*:

(3) $$p^{2}=\frac{\omega^{2}}{c_{L}^{2}}-k^{2}$$

(4) $$q^{2}=\frac{\omega^{2}}{c_{T}^{2}}-k^{2}$$

The velocities of shear and longitudinal waves in an infinite medium denoted as $c_{T}$ and $c_{L}$ can be calculated based on known material parameters of the medium:

(5) $$c_{L}=\sqrt{\frac{\lambda +2\mu }{\rho }}$$

(6) $$c_{T}=\sqrt{\frac{\mu }{\rho }}$$

where *µ* and *λ* are Lame’s constants. The group velocity is the derivative of angular frequency over the wavenumber:

(7) $$c_{g}=\frac{d\omega }{dk}$$

For the given angular frequency, there is an infinite number of possible solutions that fulfill Equations (1) and (2). The wavenumber *k* can be real, imaginary, or complex; however, if the plate is considered unloaded, it is sufficient to consider real values only. The number of possible solutions also increases with frequency range. For higher frequencies, the number of possible wave modes increases.

### 2.2. Two-Dimensional Fourier Transform

The shape of dispersion curves can be determined by solving the dispersion equations, but also by processing the signals captured at the investigated structure. It is possible to measure the amplitudes of individual Lamb wave modes by using a 2-dimensional fast Fourier transform (2D-FFT) technique [21]. In this approach, the time-domain propagation signals recorded at the series of equally spaced positions along the propagation path are transformed and the data from the time–space plane are converted into the frequency–wavenumber plane according to the following expression:

(8) $$Y(k,\omega )=\int_{-\infty }^{+\infty }\int_{-\infty }^{+\infty }u(x,t)e^{-i(kx+\omega t)}dxdt$$

where **u**(*x*,*t*) denotes the displacement of the surface.

## 3. Concrete Model Generation

### 3.1. Concrete Mesostructure

Concrete is a multi-phase, strongly heterogeneous material and its mesostructure presents strong randomness characteristics. It consists of aggregates varying in diameter and shape, mortar mix, interface transition zones (ITZs) located between mortar and aggregates, cracks, and pores. All concrete components are randomly distributed within its volume. The major part of the concrete mixture is aggregate particles. They can be divided into two groups by size: Fine (≤4.75 mm) and coarse aggregate (>4.75 mm). The content of coarse aggregate in the concrete volume is usually equal to 40–50% and, thus, it largely determines the parameters of the concrete mixture, as well as its cost. The particle shape depends on the aggregate type. However, after other researchers [17,19,22] and for the sake of simplicity, in a later part of the paper, we assume that they are spherical.

The next component distinguished at the mesoscopic level is ITZs, which are the regions of the cement film covering the aggregate particles. As the first micro-cracks are induced at the interface, the ITZs are considered as the weak link in the concrete. Their negligible influence on guided wave propagation velocity was presented by Xu et al. [17] and, therefore, their existence is omitted in further investigations.

In this study, we consider a two-phase concrete model comprising mortar matrix and aggregate particles. In the following sections, the process of model generation is described step by step. The mesostructure of the concrete is generated using the Monte Carlo method, which allows for generation of random numbers with a specific distribution. The particles’ diameters are chosen based on the a priori known grading curve, and particles’ coordinates are generated by taking into account the certain restrictions related to the model dimensions.

### 3.2. Aggregate Particles Size

**Figure 2:**
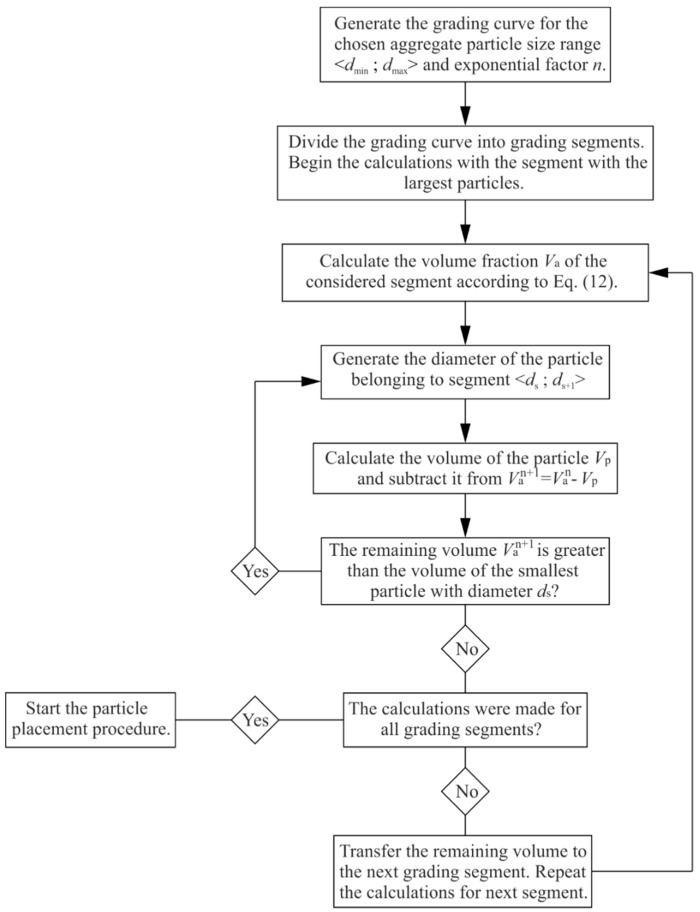
Generation of the aggregate particles.

This section discussed the developed algorithm of diameter generation of the particles (Figure 2). The particle size distribution in concrete is expressed in terms of cumulative percentage passing through a series of sieves with openings with different sizes. The contribution of particle size fractions is usually presented in the form of a grading curve. The most popular and commonly used grading curve, which lies within the limiting grading curves proposed by design recommendations [23], is the empirical curve proposed by Fuller [22,24]. Fuller’s curve provides optimum density and strength of the concrete and is described by the following formula:

(9) $$P(d)={\left(\frac{d}{d_{\max}}\right)}^{n}\cdot 100\%$$

where *P*(*d*) is the cumulative percentage passing through the sieve with diameter opening *d*, *d*_max_ is the maximum particle size, and *n* is the exponential factor with a typical value of 0.4–0.7. The weight percentage of the individual grading segment can be calculated as

(10) $$P\left[d_{s},d_{s+1}\right]=P(d_{s})-P(d_{s+1})$$

The aggregate volume fraction in the concrete mixture is

(11) $$v_{a}=\frac{m_{a}}{\rho_{a}V}$$

where *m_a_* is the mass of aggregate particles, *ρ_a_* is aggregate material density, and *V* is the volume of the concrete specimen. The volume of the particles in the grading segment $V_{a}\left[d_{s},d_{s+1}\right]$ can then be calculated as

(12) $$V_{a}\left[d_{s},d_{s+1}\right]=\frac{P(d_{s})-P(d_{s+1})}{P(d_{\max})-P(d_{\min})}\cdot v_{a}\cdot V$$

To generate the particle belonging to the grading segment with randomly chosen diameter, one can use the formula:

(13) $$d_{p}=\eta \left(d_{s+1}-d_{s}\right)+d_{s}$$

where *η* is a uniformly distributed random number in the interval (0,1). The number of particles generated in this way must ensure that the difference between the sum of their volumes and the volume $V_{a}\left[d_{s},d_{s+1}\right]$ is smaller than the volume of the smallest particle vs. belonging to the considered segment:

(14) $$V_{a}[d_{s},d_{s+1}]-\sum_{i}^{n}V_{p}^{i}>V_{s}$$

Based on the described relationships, the algorithm of diameter generation of the particles was performed.

### 3.3. Particle Placement

**Figure 3:**
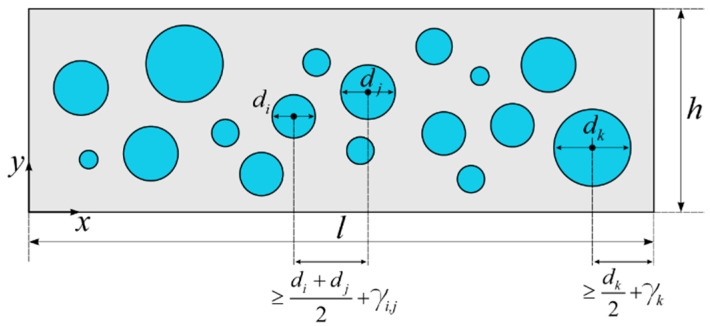
Particle placement in concrete specimen.

Next, the generated particles must be placed into the concrete volume. As mentioned, they are randomly distributed; however, their placement must satisfy some primary conditions. First, all particles must be located within the concrete volume, and secondly, none of them can overlap with each other. Moreover, the placement process should include that the distance *s*_*i*,*j*_ between the centers of adjacent particles must be greater than the sum of their radii because of the mortar film covering the aggregates (Figure 3):

(15) $$s_{i,j}\geq \frac{d_{i}+d_{j}}{2}+\gamma_{i,j}$$

In this study, the thickness of mortar film is equal to 5% of the sum of the diameters of adjacent particles [24]:

(16) $$\gamma_{i,j}=0.05\cdot \left(d_{i}+d_{j}\right)$$

Additionally, the distance between the particle and the specimen boundary must be at least equal to:

(17) $$\gamma_{i}=0.05\cdot d_{i}$$

The above conditions imply that the coordinates of the mass center of the particle with diameter *d* located within the volume of the concrete plate with thickness *h* and length *l* must be generated in the following way:

(18) $$x_{i}=\eta \left(x_{\max}-x_{\min}\right)+x_{\min}=\eta \left(l-1.1d_{i}\right)+0.55d_{i}$$

(19) $$y_{i}=\eta \left(y_{\max}-y_{\min}\right)+y_{\min}=\eta \left(h-1.1d_{i}\right)+0.55d_{i}$$

where *η* is the uniformly distributed random number in the interval (0,1).

The algorithm of the particle placement can be summarized in three steps:

- Step I: For the particle with diameter *d_i_*, generate the coordinates of its mass center. It is recommended to begin from the largest particles, which significantly facilities the placement process.
- Step II: Check if the particle covered with the mortar film does not overlap with any other previously generated particle. If it does, generate new coordinates and check again. If particles do not overlap, generate the coordinates for the next particle.
- Step III: Repeat Steps I and II for all particles.

### 3.4. Description of Numerical Models

#### 3.4.1. Model Geometry and Material Parameters

**Figure 4:**
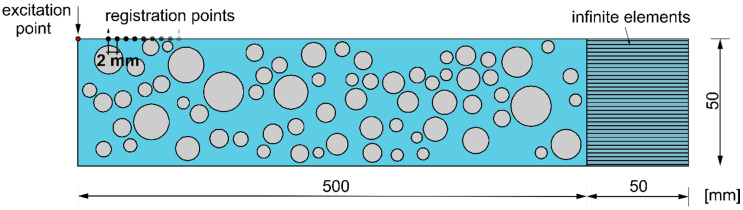
Geometry of numerical models.

**Figure 5:**
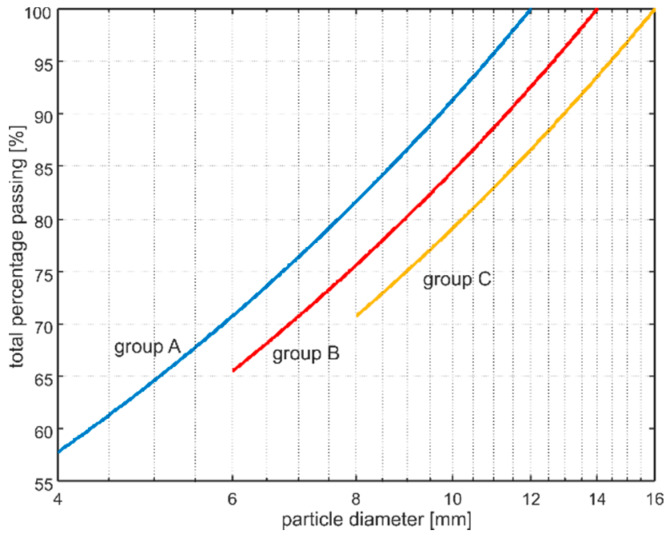
Fuller’s curves for concrete models considered in the study.

The plate model developed in the Abaqus/Explicit environment has the dimensions 50 mm × 500 mm (Figure 4). The adopted material parameters of the mortar matrix and aggregate are summarized in Table 1 [17,25]. The calculations were performed for nine models varying in aggregate ratio and aggregate particle size (Table 2). The grading curves for particular models are presented in Figure 5. In all cases, the exponential factor *n* was equal to 0.5. Particles smaller than 4 mm in diameter were not introduced in any model, to avoid the problems with mesh generation.

**Figure 6:**
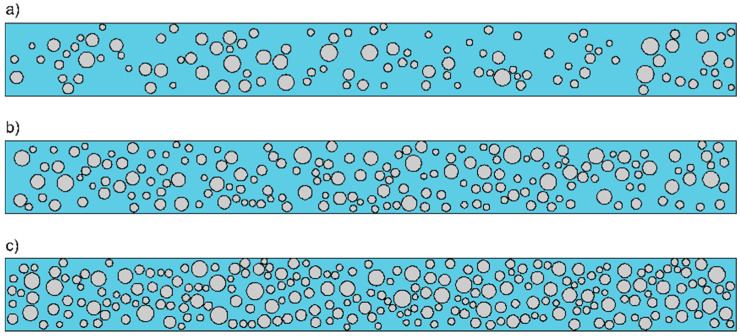
Geometry of numerical models varying in aggregate ratio: (**a**) Model A1, (**b**) model A2, and (**c**) model A3.

**Figure 7:**
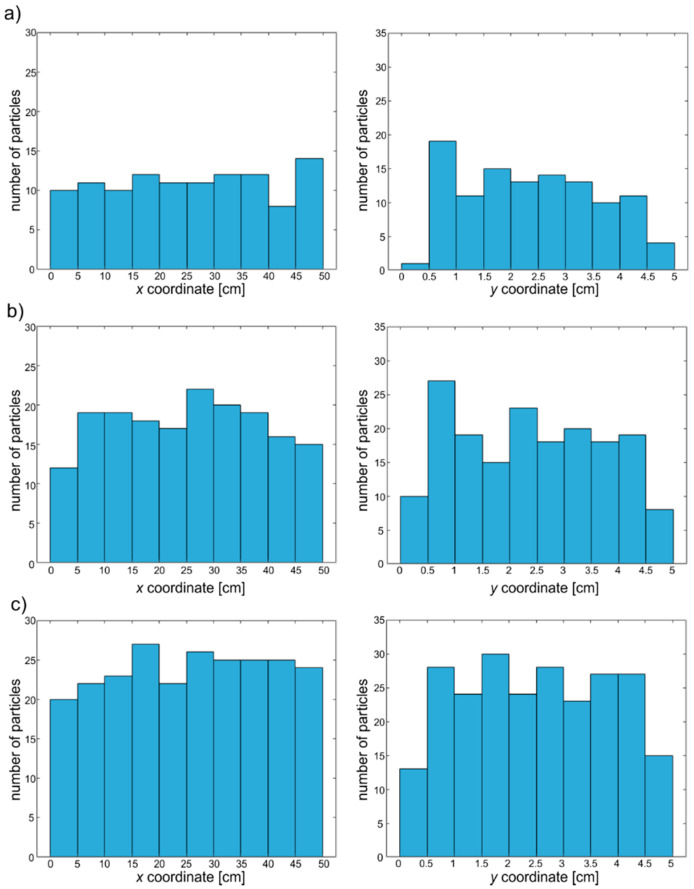
Histograms of the special distribution of the generated particles for model (**a**) A1, (**b**) A2, and (**c**) A3.

The models were developed with the use of four-node plane strain elements with reduced integration (CPE4R). To ensure that the measured signals are dominantly influenced by the concrete mesostructure, a layer of four-node infinite plane strain elements (CINPE4) with a thickness of 50 mm was additionally introduced. The layer of infinite elements allows us to avoid the registering of reflection from the edge of the plate. Moreover, they allow the size of the investigated structure to be reduced. The dimensions of the finite elements were 1 mm × 1 mm and the length of the integration step was to Δ*t* = 10^−8^ s. The dimensions of the infinite elements were 1 mm × 50 mm. To visualize the differences in aggregate particle concentration, the geometries of three exemplary models are presented in Figure 6. Additionally, Figure 7 shows histograms of the special distribution of the generated particles for three different aggregate ratios. It can be seen that the distribution is more or less uniform, which confirms the random generation of particle placement.

#### 3.4.2. Excitation Function

The excitation function was in the form of a windowed tone burst, which is commonly used in Lamb wave-based inspections [26]. The tone burst is a modulated sine or cosine function by the Hann or Gaussian window. The modulation allows the dispersion effect to be reduced and provides the mode purity [26]. In this study, the five-cycle sine function with carrier frequencies of 25, 50, 100, and 150 kHz modulated by the Hann window was used. In the numerical model, the excitation was applied as a concentrated time-dependent force. As, in the actual cases, the actuators and sensors are usually attached at the surface of the plate, the excitation was applied perpendicularly to the plate surface. The signals were registered at the series of positions spaced 1 mm apart along the propagation path with a length of 49.5 cm.

## 4. Results

### 4.1. Visualization of Lamb Wave Propagation

**Figure 8:**
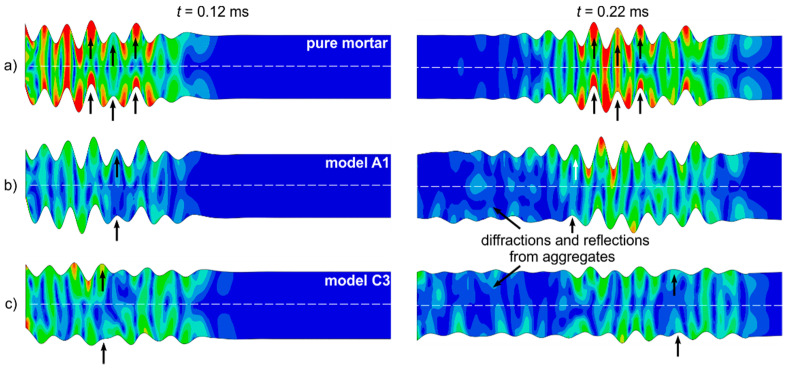
Visualization of wave propagation in (**a**) homogeneous concrete plate, (**b**) heterogeneous model A1, and (**c**) heterogeneous concrete model C3.

Visualization of the propagating wave in the form of magnitudes of displacements at selected time instants is presented in Figure 8. For comparison, the figure also contains the results for a homogeneous plate characterized by mortar parameters (aggregate ratio 0%). It can be seen that in the case of the homogeneous plate, the displacements of outer surfaces are the same and the map is symmetrical with respect to the middle plane, which clearly indicates the existence of an antisymmetric mode. The presence of an even smaller number of aggregate particles significantly affects the observed wave motion and mode purity. The displacements of the outer surfaces in model A1 are not the same. Moreover, the symmetry of the displacement map is not sustained (Figure 8b). The symmetry disruption becomes more visible in the model with a higher aggregate ratio (Figure 8c). In the case of heterogeneous models, the propagating wave reflects from the particles characterized by different material parameters than the mortar matrix. Part of the reflected wave energy propagates back along the plate model. Note that, in the case of the homogeneous model after 0.22 ms, wave motion is not observed at the initial part of the model, while in heterogeneous models, low-amplitude wave motion resulting from wave interactions with particles is observed in the entire plate volume. It is noteworthy that additional wave phenomena like diffractions and reflections resulting in additional peaks registered in signals may lead to significant difficulties in interpretation of the results.

The aggregate ratio also affected the dissipated energy. The wave amplitude is clearly higher for the model made of pure mortar. The displacements caused by wave motion becomes lower for increasing aggregate ratio.

### 4.2. Dispersion Curves

**Figure 9:**
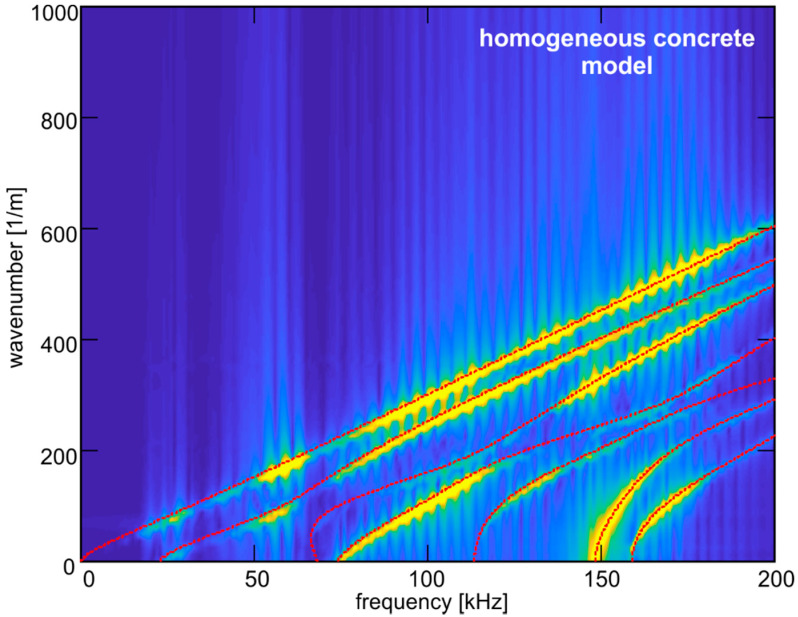
Wavenumber–frequency representation for homogeneous concrete model.

**Figure 10:**
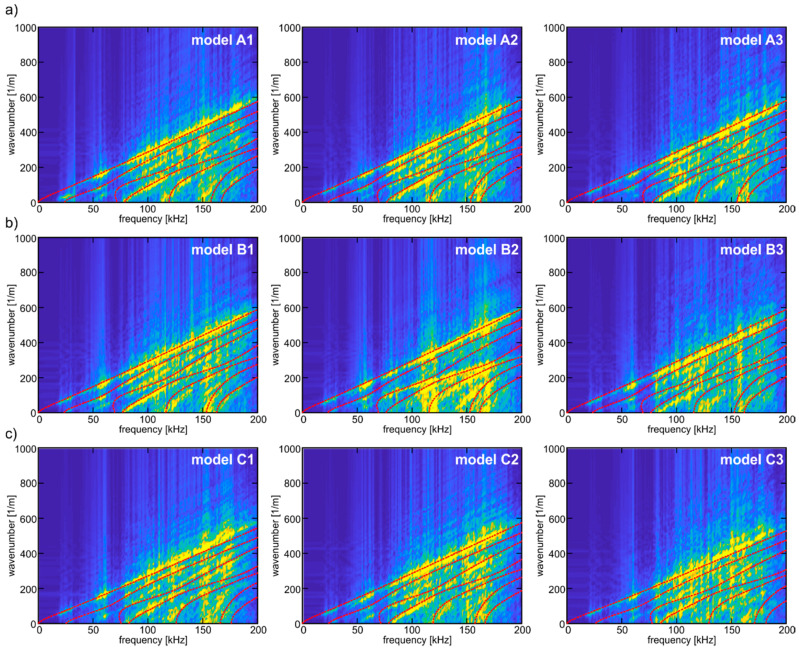
Wavenumber–frequency maps determined for numerical models of heterogeneous concrete plates: (**a**) group A; (**b**) group B and (**c**) group C.

Figure 9 and Figure 10 present the 2-D view of the wavenumber–frequency information, which was obtained by carrying out the 2D-FFT (see Equation (8)) of the time series registered for four different excitation frequencies in equally spaced points on the plate surface. The final map presents the dispersion curves representing antisymmetric Lamb wave modes described by Equation (2). Based on the 2D-FFT results, the shape of the curve for the first antisymmetric mode was reconstructed. For comparison, the wavenumber–frequency information obtained for the homogeneous concrete model is presented in Figure 9, while Figure 10 contains the results for nine heterogeneous models.

As we can see, the aggregate ratio clearly influences the visibility of the particular dispersion curves. In the case of the homogeneous material, the curves can be unambiguously distinguished. The presence of aggregate particles and aggregate-wave interactions affecting the signals’ characteristics resulted in deterioration of the quality of the maps. The map with the worst quality was obtained for model C3 characterized by the highest aggregate ratio and the largest particles (see Table 2). Heterogeneity hinders the reconstruction of dispersion curves based on wavenumber–frequency information.

### 4.3. Determining the Elastic Modulus of Concrete Plates

To compare the numerical results with theoretical predictions, the elastic modulus was estimated in two ways. First, the shape of dispersion curves was reconstructed based on numerical results. Secondly, it was calculated based on known proportions between aggregate particles and mortar matrix.

The elastic modulus identification procedure was developed in a MATLAB environment using function fminsearch. The procedure of curve shape reconstruction can be summarized in four steps. In the first step, the data obtained from the FEM analysis were transformed in the wavenumber–frequency domain using a 2D-FFT-based algorithm. Further considerations were conducted for the limited representative area of data (Figure 10a). In our case, due to the use of excitation in the form of wave packets with central frequencies of 25, 50, 100, and 150 kHz, the frequency range, which was taken into account, was 0–250 kHz and the corresponding wavenumber range was 0–1000 m^−1^. To optimize the calculation process, the 2D-FFT results were normalized with respect to the maximum amplitude value for each frequency:

(20) $$\hat{Y}(k_{i},\omega_{i})=\frac{Y(k_{i},\omega_{i})}{\max\left\{Y(\omega_{i})\right\}}$$

**Figure 11:**
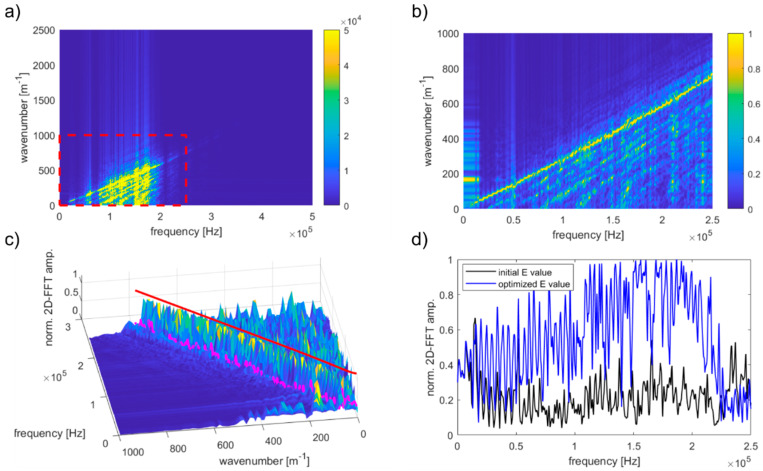
The Young modulus determining algorithm: (**a**) The 2D-FFT map and the data area selected for further analysis; (**b**) results of the normalization; (**c**) visualization of 2D-FFT interpolation along the dispersion curve; (**d**) comparison of interpolation results for a pre-selected and finally appointed Young modulus values.

The normalization process excluded the unequal influence of the individual frequencies. Moreover, there was no need to use weight functions. Examples of normalized data are shown in Figure 11b. Normalization was the last stage of data preparation. In the next step, the procedure for determining the elastic modulus was initiated. In the first step, the dependence for the first antisymmetric Lamb mode was calculated for the pre-established Young modulus. The determined values creating a dispersion curve were considered as the sets of *n* pairs of numbers:

(21) $$K=\left\{\left(\Delta \omega ,k(\Delta \omega )\right),\left(2\Delta \omega ,k(2\Delta \omega )\right),\ldots ,\left(n\Delta \omega ,k(n\Delta \omega )\right)\right\}=\left\{\left(\Delta \omega ,k_{1}\right),\left(2\Delta \omega ,k_{2}\right),\ldots ,\left(n\Delta \omega ,k_{n}\right)\right\}$$

Next, the standardized 2D-FFT values for particular angular frequencies were interpolated, forming a second set of data (Figure 10c,d):

(22) $${\hat{Y}}_{\mathrm{int}}=\left\{\hat{Y}\left(\Delta \omega ,k_{1}\right),\hat{Y}\left(2\Delta \omega ,k_{2}\right),\ldots ,\hat{Y}\left(n\Delta \omega ,k_{n}\right)\right\}=\left\{{\hat{Y}}_{1},{\hat{Y}}_{2},\ldots ,{\hat{Y}}_{n},\right\}$$

Finally, the value of the following function was calculated:

(23) $$\hat{F}=\frac{1}{n\sum_{i}^{n}{\hat{Y}}_{i}^{2}}$$

The procedure was repeated for different values of elastic moduli until the function $\hat{F}$ reaches a minimum value. The minimum value of the function $\hat{F}$ indicated that the dispersion curve that analytically determined the best coincides with the dispersion curve visible in the map. This procedure was tested first for the homogeneous material with parameters *E* = 26 GPa, *v* = 0.2, and *ρ* = 2100 kg/m^3^. The elastic modulus determined based on numerical results was 25.92 GPa and the percentage error was 0.309%, which is a satisfactory consistency of results. The analytical dispersion curves for the finally determined Young modulus were imposed on the numerical maps by red dashed lines (Figure 10), while the corresponding values of Young’s modulus denoted as $E_{c}^{DC}$ are summarized in Table 3.

The second stage of the analysis involved the theoretical calculation of Young’s modulus. There are several theoretical models, which allow the elastic modulus of two-phase heterogeneous materials to be estimated. Two most common approaches were proposed by Voigt and Reuss. The use of either of these two models requires knowledge of the modulus of elasticity of mortar and aggregate and the volume of aggregates. According to the Reuss model, the elastic modulus is calculated in the following way:

(24) $$E_{C}^{R}=\frac{E_{m}}{1+\left(\frac{E_{m}}{E_{a}}-1\right)V_{a}}$$

The overall elastic modulus of concrete by Voigt is:

(25) $$E_{C}^{V}=E_{m}\left(1+\left(\frac{E_{a}}{E_{m}}-1\right)V_{a}\right)$$

where *E_m_* and *E_a_* are elastic moduli of mortar and aggregate, respectively, and *V_a_* is the volume fraction of aggregate. The elastic modulus calculated for all nine models is summarized in Table 3. Additionally, the result for the homogeneous concrete model was added for comparison. It can be seen that if *E_a_* > *E_m_*, the Voigt model always predicts higher values of the elastic modulus than the Reuss model. The experimental study presented in previously published papers showed that these two models usually define the upper and lower bound of the concrete elastic modulus and the exact value usually lies between their predictions [27]. Indeed, the modulus value predicted on the basis of dispersion curves $E_{c}^{DC}$ lies between theoretically determined boundary values. The differences between particular results, as well as the percentage errors, are reported in Table 4.

It can be seen that Young’s modulus calculated according to both theoretical models clearly increased with the volume fraction of aggregate particles. The results obtained using the dispersion curves do not show the same increasing tendency. As expected, the elastic modulus was found to be the highest for models A3, B3, and C3, but the value of $E_{c}^{DC}$ was higher for model A1 than for A2, and that for model C1 was higher than that for C2. This means that the aggregate presence affected wave propagation velocity. Moreover, the wave-aggregate interactions could affect the signal characteristics, which, in turn, resulted in a change in the shape of the dispersion curves.

Comparing the values of percentage errors reported in Table 4, it is clearly visible that the theoretical Reuss models are better suited to numerical results obtained by dispersion curves’ reconstruction. The average percentage error for the Reuss model is 5.714%, while for the Voigt model, it is 9.633%. The greatest differences were reported for models with an aggregate ratio of 40%: 8.614% for A3, 9.636% for B3, and 13.943% for C3. One can conclude that the discrepancy between theoretical and experimental results clearly increases with the number of scatterers, but also with their size.

**Figure 12:**
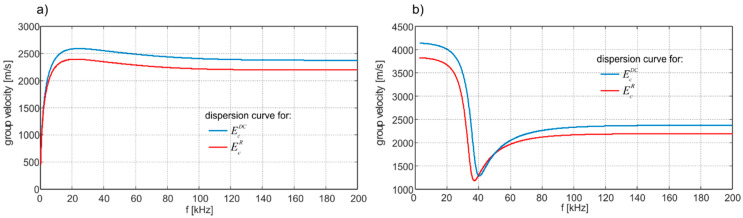
Comparison of (**a**) antisymmetric and (**b**) symmetric modes traced for various values of Young’s modulus.

The presented results indicate that the heterogeneity of concrete influences wave propagation characteristics. The elastic modulus estimated using most common theoretical models differ from the modulus estimated based on dispersion curves. Meanwhile, the difference in modulus values leads to discrepancies in wave velocity estimation, which is particularly important if the wave velocity is used as an indicative parameter in the diagnostic process. To illustrate the differences in wave velocity, the Lamb dispersion equations were solved for model C3, for which the highest error was noted. Figure 12 contains a comparison of the first symmetric and antisymmetric modes. Disregarding the impact of the concrete mesostructure may lead to incorrect velocity determination—for some frequencies, the discrepancy may reach over 400 m/s.

## 5. Conclusions

The paper presents the results of the investigation of the influence of a concrete mesostructure on Lamb wave propagation. The numerical calculations of guided wave propagation were performed for two-phase concrete plate models varying in aggregate ratio and particle diameter size. The random distribution of aggregate particles was generated using the Monte Carlo method. The diameter size was generated according to Fuller’s grading curve.

The numerical visualization showed that the heterogeneous mesostructure of the concrete plate affected the displacements associated with wave motion. The displacement maps were symmetric with respect to the middle plane only for a homogeneous concrete plate. The presence of even a small number of particles disrupted the displacement symmetry, as well as the wave amplitude.

The study also involved the comparison of Young’s modulus determined in two different ways: Theoretically based on two models commonly used for two-phase heterogeneous materials (Reuss and Voigt model) and numerically by reconstructing the dispersion curves calculated with the use of time-domain signals registered at the plate surface after wave excitation. The values of elastic modulus obtained theoretically and numerically differ, and the discrepancy increased with aggregate ratio and the size of aggregate particles. The results obtained clearly indicate that a Lamb wave propagating in an isotropic, homogeneous material characterized by the averaged macroscopic parameters is characterized by different velocities than the wave propagating in a heterogeneous material. The discrepancy increased with an increasing number of scatterers associated with more often wave-aggregate interactions, affecting wave propagation. The influence of wave-aggregate interactions also increased with particle size as the diameter approached the wavelength. These observations are particularly important for diagnostic procedures, which use wave propagation velocity as an indicative parameter for structural state assessment.

## Figures and Tables

**Table 1:** Material parameters of matric mortar and aggregate particles.

Material Parameter	Mortar Matrix	Aggregate Particles
Elastic modulus (GPa)	26	60
Poisson’s ratio (-)	0.2	0.22
Density (kg/m^3^)	2100	2700

**Table 2:** Parameters of numerical models.

Model	Aggregate Ratio	Aggregate Particle Size (mm)
A1	20%	
A2	30%	4–12
A3	40%	
B1	20%	
B2	30%	6–14
B3	40%	
C1	20%	
C2	30%	8–16
C3	40%	

**Table 3:** Parameters of numerical models.

Model	Aggregate Volume Fraction (-)	Density (kg/m^3^)	$E_{c}^{V}$ acc. Voigt Model (GPa)	$E_{c}^{R}$ acc. Reuss Model (GPa)	$E_{c}^{DC}$ Based on Dispersion Curves (GPa)
homogeneous concrete model	0	2100	26	26	25.92
A1	0.163	2197.8	31.542	28.650	30.310
A2	0.250	2250.0	34.500	30.290	29.459
A3	0.342	2305.2	37.628	32.250	35.290
B1	0.163	2197.8	31.542	28.650	28.734
B2	0.250	2250.0	34.500	30.290	29.119
B3	0.342	2305.2	37.628	32.250	35.689
C1	0.163	2197.9	31.542	28.650	29.400
C2	0.250	2250.0	34.500	30.290	29.104
C3	0.342	2305.2	37.628	32.250	37.475

**Table 4:** Differences between theoretical and numerical results.

Model	The Difference $\left|E_{c}^{V}-E_{c}^{DC}\right|$ (GPa)	Percentage Error $\frac{\left|E_{c}^{V}-E_{c}^{DC}\right|\cdot 100\%}{E_{c}^{DC}}$ (%)	The Difference $\left|E_{c}^{R}-E_{c}^{DC}\right|$ (GPa)	Percentage Error $\frac{\left|E_{c}^{R}-E_{c}^{DC}\right|\cdot 100\%}{E_{c}^{DC}}$ (%)
homogeneous concrete model	0.08	0.309	0.08	0.309
A1	1.232	4.065	1.66	5.477
A2	5.041	17.111	0.831	2.820
A3	2.338	6.625	3.040	8.614
B1	2.808	9.772	0.084	0.292
B2	5.381	18.479	1.171	4.021
B3	1.939	5.433	3.439	9.636
C1	1.842	6.265	0.750	2.551
C2	5.396	18.540	1.186	4.075
C3	0.153	0.408	5.225	13.943
